# Assessment of clinical and epidemiological characteristics of patients with and without sepsis in intensive care units of a tertiary hospital

**DOI:** 10.31744/einstein_journal/2019AO4476

**Published:** 2019-03-29

**Authors:** Luis Gustavo Morello, Líbera Maria Dalla-Costa, Rafael Mialski Fontana, Ana Cristina Schmidt de Oliveira Netto, Ricardo Rasmussen Petterle, Danieli Conte, Luciane Aparecida Pereira, Marco Aurélio Krieger, Sonia Mara Raboni

**Affiliations:** 1 Instituto Carlos Chagas, Fundação Oswaldo Cruz, Curitiba, PR, Brazil.; 2 Hospital de Clínicas, Universidade Federal do Paraná, Curitiba, PR, Brazil.

**Keywords:** Blood culture, Sepsis/diagnosis, Bacterial infections, Critical care, Hemocultura, Sepse/diagnóstico, Infecções bacterianas, Cuidados intensivos

## Abstract

**Objective:**

To describe the clinical and epidemiological features of patients with and without sepsis at critical care units of a public hospital.

**Methods:**

A cross-sectional study was carried out from May 2012 to April 2013. Clinical and laboratory data of patients with and without sepsis in the intensive care units were reviewed of medical records.

**Results:**

We evaluated 466 patients, 58% were men, median age was 40 years, and 146 (31%) of them were diagnosed with sepsis. The overall mortality was 20% being significantly higher for patients with sepsis (39%). The factors associated with intensive care unit mortality were the presence of sepsis (OR: 6.1, 95%CI: 3.7-10.5), age (OR: 3.6, 95%CI: 1.4-7.2), and length of hospital stay (OR: 0.96, 95%CI: 0.94-0.98). Pulmonary (49%) and intra-abdominal (20%) infections were most commonly identified sites, and coagulase-negative staphylococci and enteric *Gram* negative bacilli the most frequent (66%) pathogens isolated.

**Conclusion:**

Although the impact of sepsis on mortality is related to patients’ clinical and epidemiological characteristics, a critical evaluation of these data is important since they will allow the direct implementation of local policies for managing this serious public health problem.

## RESUMO

**Objetivo:**

Descrever as características clínicas e epidemiológicas de pacientes com sepse e sem sepse em unidades de cuidados intensivos de um hospital público.

**Métodos:**

Estudo transversal realizado de maio de 2012 a abril de 2013. Os dados clínicos e laboratoriais de pacientes com sepse e sem sepse das unidades de terapia intensiva foram revisados a partir dos prontuários médicos.

**Resultados:**

Avaliamos 466 pacientes, 58% homens, mediana de idade 40 anos; sendo 146 (31%) diagnosticados com sepse. A mortalidade global foi 20%, e significativamente maior para pacientes com sepse (39%). Os fatores associados à mortalidade em unidade de terapia intensiva foram a presença de sepse (OR: 6,1, IC95%: 3,7-10,5), idade (OR: 3,6, IC95%: 1,4-7,2) e tempo de internação (OR: 0,96, IC95%: 0,94-0,98). As infecções pulmonares (49%) e intra-abdominais (20%) foram os focos mais comumente identificados, e os estafilococos coagulase-negativa e bacilos entéricos *Gram*-negativos foram os patógenos isolados mais frequentes (66%).

**Conclusão:**

Embora o impacto da sepse sobre a mortalidade esteja relacionado às características clínicas e epidemiológicas dos pacientes, uma avaliação crítica desses dados é importante, pois permitirá a implementação direta de políticas locais para gerenciar este grave problema de saúde pública.

## INTRODUCTION

Sepsis is defined as “life threatening organ dysfunction caused by a dysregulated host response to infection”.^(^ ^1^ ^)^ With the technical improvements in Advanced Life Support in the last few years, more patients presented with sepsis. In Brazil, sepsis is responsible for nearly 13% of all intensive care unit (ICU) admissions, and the number of deaths due to sepsis has increased by approximately 6%, from 2000 to 2010.^(^ ^2^ ^-^ ^4^ ^)^ Data from the *Instituto Latino Americano da Sepse* (ILAS) show that sepsis-related mortality rate, at Brazilian private and public hospitals, ranges from 30% to 70%, respectively.^(^ ^5^ ^,^ ^6^ ^)^ Factors related to mortality include time to initiate on antibiotics, infection control, and fluid infusion, in addition to factors intrinsic to patients, such as age and comorbidities.^(^ ^7^ ^)^

Blood culture is the gold standard technique for microbiological diagnosis to confirm infection and indicate the appropriate antimicrobials required for treatment.^(^ ^1^ ^)^ Although its low sensitivity and longer turnaround time, it is the method that is available in most hospitals. Molecular methods are very sensitive and rapid for etiologic diagnosis; however, their applicability in clinical settings is still limited.^(^ ^8^ ^)^

In Brazil, information on the clinical impact of sepsis in public hospitals, as well as data on the prevalence of microorganisms associated with severe infections in ICU are scarce. In this study we describe the clinical, epidemiological, and microbiological findings of patients admitted to ICU of a Brazilian public tertiary hospital, and compare the data from patients with and without sepsis.

## OBJECTIVE

To describe the clinical and epidemiological characteristics of patients with sepsis and without sepsis, in critical care units.

## METHODS

A cross-sectional study was carried out from May 2012 to April 2013. Data were obtained retrospectively from the medical records of the Hospital Information System of the *Hospital de Clínicas* of *Universidade Federal do Paraná* (UFPR). The hospital is a 593-bed public teaching tertiary care organization with 5 ICU comprising 78 beds and divided into adult, adult stepdown, cardiac, pediatric, and neonatal units. The Research Ethics Committee of the organization approved this study (CAAE: #03377612.5.0000.0096).

During the study period, there were 1,767 admissions to the 5 above-mentioned ICU. Considering a 95% confidence interval (95%CI) and a sample error rate of 5%, and setting the response distribution to 50% (the most conservative assumption), the minimum representative sample size was estimated at 316 admissions.^(^ ^9^ ^)^ Accordingly, we randomly included 466 ICU admissions, which corresponded to 26% of the total admissions. The admissions were designated as sepsis or non-sepsis cases, based on the discharge summaries. Data such as age, ICU setting, the length of hospital stay, presence of sepsis, severity of sepsis (septic shock), infection site, and outcome (death or discharge) were evaluated. In parallel, the positivity rate of blood cultures and the isolated microorganisms were also evaluated.

Sepsis was defined as cases of infection with systemic repercussions (meeting at least two of the following criteria: core body temperature >38°C or <36°C, heart rate ≥90bpm, respirations ≥20/minute, and/or carbon dioxide parcial pressure PaCO_2_ <32mmHg). Severe sepsis was defined as cases of sepsis-related organ dysfunction (renal dysfunction, pulmonary dysfunction – acute respiratory distress syndrome, hematologic or circulatory dysfunction responsive to fluid therapy). Septic shock was defined as cases of hypotension refractory to fluid resuscitation, requiring vasoactive drugs, and presence of hyperlactatemia. It is important to highlight that the study was designed using the definitions of sepsis and septic shock revised in 2001.^(^ ^10^ ^)^ The updated definitions reported by Singer et al.,^(^ ^1^ ^)^ were published after the data collection of the present study.

### Statistical analysis

Data were analyzed using both GraphPad Prism version 5.03 (GraphPad Software, Inc., La Jolla, CA, USA) and R version 3.1.2 (R Core Team, 2014). Continuous variables are represented as median values with interquartile ranges (IQR). Group comparisons were performed using the χ^2^ or Fisher’s exact test for categorical variables, and Mann-Whitney test for continuous variables, as appropriate. A 95%CI was adopted, and p values <0.05 were considered statistically significant. Kaplan-Meier survival curves for pediatric and adult patients, and for patients with and without sepsis were constructed and compared by a log-rank test.

To determine the independent contribution of the study variables to ICU death, possible explanatory variables − ICU unit (pediatric or adult) and the presence of sepsis − were included in a logistic regression model. The final multivariate logistic regression model included only the factors that remained independently, and were significantly associated with outcome during hospital stay, after adjustment for the effects of all other variables. When variables independently related to outcome were determined, and the predictive model was established, we calculated the association of predicted probabilities of death, and the lengths of hospitalization for this analysis were set on days 5, 11 and 24.

## RESULTS

A total of 466 admissions, corresponding to 409 patients, were evaluated, of which 58% (273) were men, and the median age was 40 years (IQR: 1-62). A total of 35.5% of admissions occurred in the pediatric ICU, while 65.5% were in the adult ICU, and sepsis was one of the complications in 146 (31%) hospitalizations. The median length of ICU stay was 11 (IQR: 5-24.5) and 4 (IQR: 1-9) days for sepsis and non-sepsis, respectively. On average, the non-sepsis group was 10 years younger than the sepsis group, and this difference was statistically significant; however, there was no significance when the neonatal ICU patients were excluded from the non-sepsis group (Table 1).

**Table 1:** Patients with and without sepsis according to the admission unit and outcomes

Parameters	Without sepsis n=320	With sepsis n=146	p value
Age, year	37 (0-61)	47 (6.5-66.5)	0.0006
Male	183 (57)	90 (61)	0.4174
Admission ICU			
Neonatal	73 (23)	23 (16)	0.0851
Pediatric	46 (15)	19 (13)	0.7739
Adult	107 (33)	62 (42)	0.0625
Adult stepdown	7 (2)	28 (19)	<0.0001^*^
Cardiac	87 (27)	14 (10)	<0.0001^*^
Outcome			
Death, ICU	36 (11)	57 (39)	<0.0001^*†^
Neonatal	5 (7)	3 (13)	0.3926
Pediatric	2 (4.3)	3 (15.8)	0.1444
Adult	20 (18.7)	33 (53)	<0.0001^*^
Adult stepdown	2 (28.5)	14 (50)	0.4150
Cardiac	7 (8)	4 (28.6)	0.0439^*^
Length of stay in ICU, days	3 (1-6)	6.5 (3-17)	<0.0001^*^
Length of stay in the hospital, days	10 (4-20)	13 (7-32)	0.0003^*^

Results expressed as median (interquartile range) or %. * Statistically significant differences; ^†^ odds ratio=5 (95%CI: 3.1-8.1).

ICU: intensive care unit.

**Figure 1:**
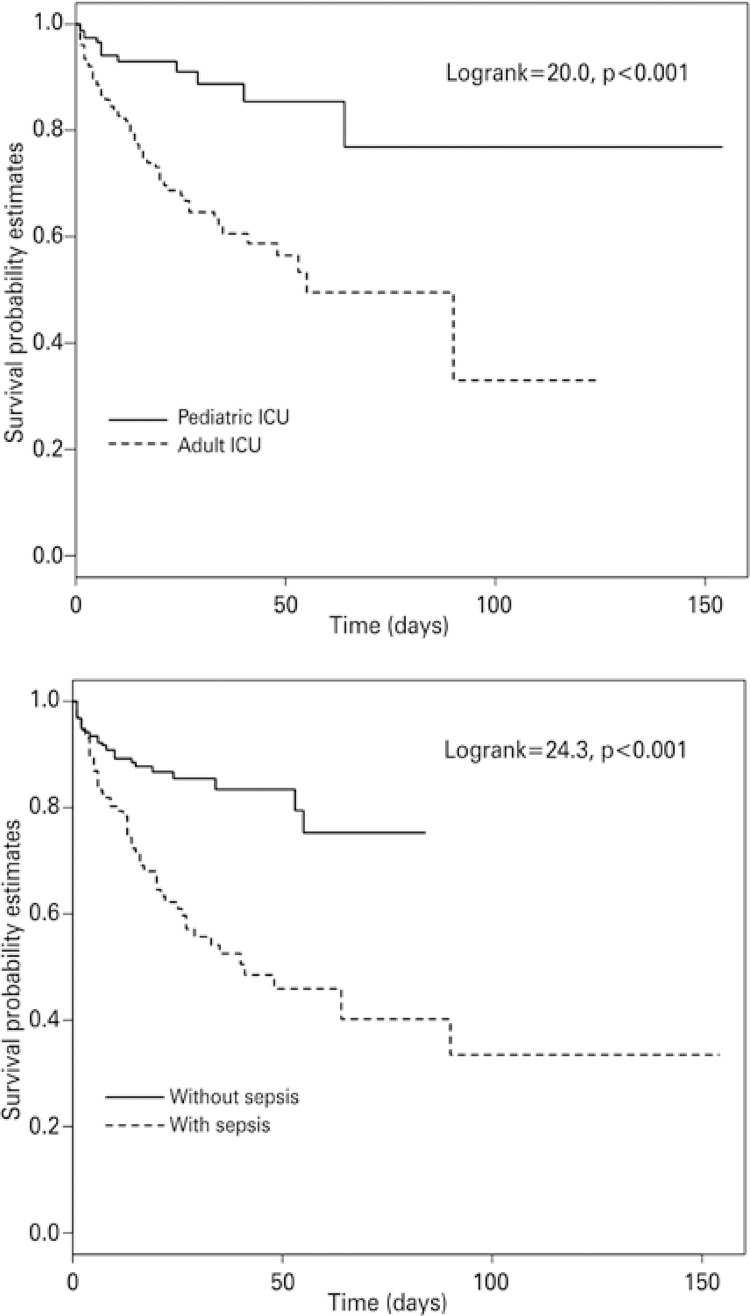

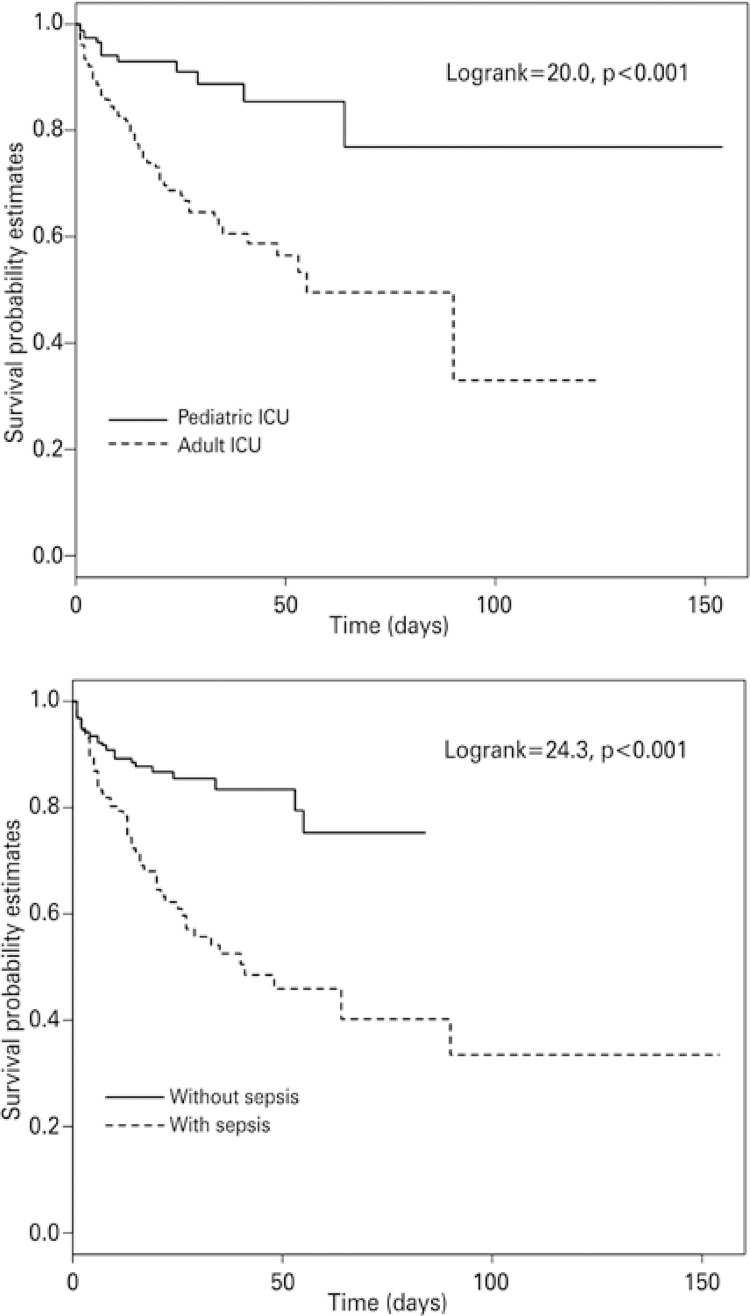
Survival analysis of pediatric and adult patients in intensive care units

The overall mortality was 20% (93 patients), and factors associated with ICU mortality in a univariate analysis were the presence of sepsis (OR: 6.1, 95%CI: 3.7-10.5), age of patients (OR: 3.6, 95%CI: 1.4-7.2), and length of hospital stay (OR: 0.96, 95%CI: 0.94-0.98). Pediatric patients and those without a diagnosis of sepsis exhibited significantly longer survival times (log-rank; p=0.001) (Figure 1). Among the patients with sepsis, 57 (39%) died. The sepsis group had a five-fold higher risk of death, and a two-fold longer ICU stay than the non-sepsis group. Furthermore, over half of the patients admitted with a diagnosis of sepsis (52%) developed septic shock, which was related to a higher risk of mortality (OR: 38, 95%CI: 12.3-116.8).

ICU: intensive care unit.

Overall, patients with sepsis admitted to the adult ICU with a shorter length of hospital stay were more prone to die in the ICU. Using the parameters from the logistic regression model and the length of ICU stay on days 5, 11 and 24, we calculated the probability values of death (Table 2). Based on this analysis we could predict, *e.g*, that a patient with sepsis in an adult ICU with a 5-day length of hospital stay would have a 60% probability of dying. In contrast, a patient without sepsis in a pediatric ICU setting with a 24-day length of stay would have a 3% probability of dying.

**Table 2:** Calculated probabilities of death based on the coefficients estimated by the logistic regression model

Critical care unit	Sepsis	Length of hospital stay (days)	Probability (%)
Pediatric	No	24	3
Pediatric	No	11	5
Pediatric	No	5	6
Adult	No	24	11
Adult	No	11	16
Pediatric	Yes	24	17
Adult	No	5	19
Pediatric	Yes	11	25
Pediatric	Yes	5	29
Adult	Yes	24	43
Adult	Yes	11	54
Adult	Yes	5	59

**Figure 2:**
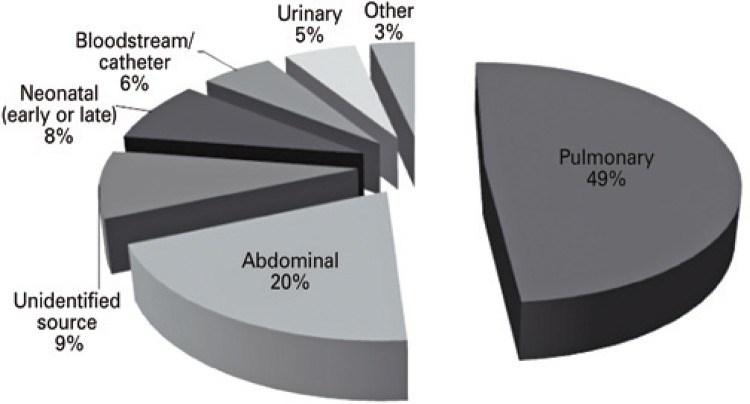
Sepsis cases according to the infection site

Nearly half of the sepsis cases were secondary to pulmonary infections (49%), followed by abdominal infections (20%), and cases of infections with unidentified sources (9%) (Figure 2). On evaluating the source of infection based on the outcomes, only abdominal infections were associated with a higher chance of septic shock, but not with higher mortality (Table 3).

**Table 3:** Sepsis cases according to infection sites and progression to shock and/or death

Infection sites	Sepsis	Septic shock	OR (95%CI)	Death	OR (95%CI)
Pulmonary	71 (50)	37 (52)	1.0 (0.5 -1.9)	26 (36)	0.8 (0.4-1.5)
Abdominal	29 (20)	22 (75)	3.6 (1.4-9.2)^**^	14 (48)	1.6 (0.7-3.6)
Unidentified source	13 (9)	9 (69)	2.2 (0.6-7.5)	9 (69)	3.9 (1.1-13.6)
Neonatal (early or late)	12 (8)	2 (17)	0.1 (0.03-0.7)^**^	2 (17)	0.2 (0.06-1.3)
Bloodstream/catheter*	8 (5)	1 (12.5)	0.12 (0.01-1.0)	2 (25)	0.5 (0.09-2.5)
Urinary	8 (5)	2 (25)	0.2 (0.05-1.4)	1 (12.5)	0.2 (0.02-1.7)
Other*	5 (3)	3 (60)	1.3 (0.2-8.6)	3 (60)	2.4 (0.4-14.9)

Results expressed as %. * Skin and soft tissues (2) and central nervous system (3). Bloodstream infection is defined as the isolation of microorganisms from blood without another identified site, and catheter-related infection is defined as the detection of the same microorganism from both the blood and catheter. ** Statistically significant differences.

OR: odds ratio; 95%CI: 95% confidence interval.

Comparing the cases with identified or presumed site of infection to those with no identified site, a significant age difference was observed. The group without an identified site of infection had a median age of 71.5 years (IQR: 25-80), while the median age was 45.5 years (IQR: 2.7-63.8) for the group with an identified infection site (p=0.0225).

**Figure 3:**
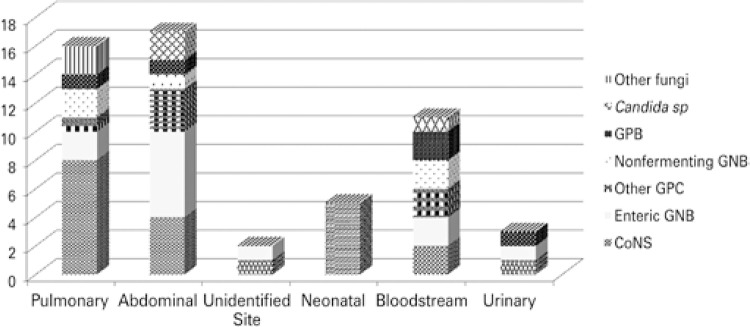
Microorganisms isolated from blood cultures according to the infection sites

GPB: *Gram* positive bactéria; GNB: *Gram* negative bacteria; GPC: *Gram* positive cocci; CoNS: coagulase-negative staphylococci.

Samples for blood cultures were collected from 195 (42%) patients admitted to the critical care units, and 18% of them were positive. Among the non-sepsis cases, blood culture samples from 89 patients (28%) were available, and 3% were positive (all coagulase-negative staphylococci). Among the sepsis cases, 73% (106) had samples collected for blood cultures, with a culture positivity of 23%. Cases of catheter/bloodstream infection had the highest culture positivity, with 40% of the collected samples testing positive. Coagulase-negative staphylococcus was the most commonly isolated pathogen, except for infections with an abdominal site, in which enteric *Gram* negative bacteria (GNB) were more frequently detected (Figure 3).

## DISCUSSION

Sepsis is a global endemic, and a major public health issue with increasing incidence.^(^ ^11^ ^,^ ^12^ ^)^ Critical analyses of factors that may be associated with sepsis, such as frequency and distribution of cases in the different ICU of our hospital, as well as time and procedures of sample collections, are reported in the present study, aiming to expand the epidemiologic data available regarding the clinical impact of sepsis in public hospitals in Brazil.

Brazilian data shows an increase of up to 40% in sepsis-related deaths in an 8-year period till 2010, predominantly in older age groups.^(^ ^4^ ^)^ The increase in life expectancy has led to a higher proportion of older patients being hospitalized due to infection, evolving to sepsis, and dying.^(^ ^4^ ^,^ ^12^ ^)^ Wang et al.,^(^ ^13^ ^)^ in a cohort of 188 North-American hospitals, showed that 60% of severe sepsis-related hospitalizations were of patients aged over 60 years. Hospitals in developing countries, especially public and teaching hospitals, tend to admit a relatively younger population into their ICU, since life expectancy in these countries is shorter and healthcare services are often insufficient.^(^ ^5^ ^,^ ^14^ ^-^ ^16^ ^)^

The age of patients evaluated in this study tended to be lower than that in other Brazilian studies; however, contrary to most epidemiological studies on sepsis, we chose to assess all hospital ICU together, and this included the neonatal and pediatric units, thereby contributing to a lower median age. Considering that only 8% admissions to the neonatal and pediatric ICU were due to sepsis,^(^ ^17^ ^)^ as opposed to 30% sepsis-related admissions to the general ICU,^(^ ^14^ ^,^ ^15^ ^)^ the age difference disappeared on excluding the neonatal and pediatric ICU admissions from both groups. Furthermore, epidemiological studies in pediatric and neonatal ICU have shown a mortality of 25%, regardless of being located in developed or underdeveloped countries,^(^ ^17^ ^)^ contrasting with the average of 50% in the adult units. Thus, in evaluating all ICU together, we may also have underestimated the overall mortality.

The advanced care units for cardiovascular diseases usually admit patients with complications, such as acute coronary syndrome and heart failure. Cases of infections in these units are generally secondary, and less common than in the general ICU. However, an increasing trend of infections in these units over the past years has been reported, with significant implication on mortality.^(^ ^18^ ^,^ ^19^ ^)^ The surgical ICU of our organization does not receive postoperative patients, and mainly admits patients in need of emergency care, thus receiving most of the complicated infection cases. The adult intensive care unit receives postoperative patients from all surgical specialties, and consequently exhibits a greater balance between sepsis and non-sepsis cases.

In addition to sepsis accounting for major costs for healthcare services, ICU deaths are also associated with increased costs compared to the survivors.^(^ ^6^ ^,^ ^20^ ^)^ Death occurred in nearly 50% of sepsis cases with longer ICU stays compared to other common diseases.^(^ ^6^ ^,^ ^10^ ^-^ ^22^ ^)^ In this study, we observed a statistically significant increased length of stay for sepsis patients. However, on outcome evaluation (death or discharge), patients who died had shorter length of hospitalization, probably a consequence of late access to ICU.

The mortality rate of 40% observed in this study is worrisome, and highlights the need to identify the factors associated with these disparities, which may be the consequence of a delay in hospitalization or in diagnosis, and inadequate therapeutic management, among others. We should also consider the possibility of a reduction in the mortality rate due to inclusion of the lower mortality rate units, such as cardiac and neonatal/pediatric ICU.^(^ ^17^ ^,^ ^19^ ^)^ In fact, analyzing only the adult critical care unit data (adult ICU and adult stepdown), the sepsis mortality observed in our cohort was similar to the 55% mortality rate reported nationally.^(^ ^5^ ^)^

Sepsis-related mortality varies greatly according to the country, organization, and even the inpatient unit.^(^ ^11^ ^,^ ^14^ ^)^ Data from Australia and New Zealand report lower mortality rates due to sepsis (<20%).^(^ ^23^ ^)^ However, within the same country, hospitals may have different rates, as shown by Wang et al.,^(^ ^13^ ^)^ in a US consortium of 188 hospitals, from 42 states, in which sepsis-related mortality ranged from 8 to 18%, depending on the hospital profile. *Instituto Latino Americano da Sepse* has the most recorded sepsis data from Brazilian hospitals, although it relies only on voluntary cooperation sites for data collection. In Brazil, within the same hospital, according to the setting (ward, emergency room, or ICU), mortality ranges from 29% to 64%. This discrepancy is also observed when comparing private and public hospitals: 34.5% of patients with sepsis die in private hospitals, while in public hospitals the average mortality rate is 55.7%.^(^ ^1^ ^,^ ^5^ ^)^

More than 25% of the suspected patients with sepsis did not have a blood sample for culture, which is a weakness in patient care. Unfortunately, this scenario is common in many clinical settings in the country, as shown by the national ILAS report, in which up to 40% of patients with sepsis did not undergo blood culture tests.^(^ ^5^ ^)^

In this study, 20% of blood cultures were positive. Some previous studies have reported a culture positivity rate of approximately 50% for emergency room^(^ ^24^ ^)^ and neonatal unit^(^ ^25^ ^)^ patients with suspected sepsis. However, usually these rates range between 10% and 30%.^(^ ^17^ ^,^ ^26^ ^,^ ^27^ ^)^ In this analysis, positive blood culture was found in 3% of the non-sepsis cases, and coagulase-negative staphylococci were detected in all these cases, which may point to a probable contamination upon collection.^(^ ^28^ ^)^ In the sepsis cases, a positivity rate of 23% was observed.

Although it is controversial whether positive blood cultures imply increased mortality,^(^ ^29^ ^)^ they are a very useful tool for both antibiotic stewardship and diagnosis of persistent infections, such as bacteremia by *Staphylococcus aureus* and *Candida* sp. The data collected retrospectively do not allow this prognostic assessment of positive blood cultures.

The lung is usually the most common infection site, which was supported by our data. The abdominal and urinary sites usually alternate as the second and third most common infection source.^(^ ^3^ ^,^ ^5^ ^,^ ^6^ ^,^ ^15^ ^,^ ^20^ ^,^ ^26^ ^,^ ^27^ ^)^ Interestingly, we identified abdominal and unidentified sources as the second and third most common sources of infection, while in neonatal ICU the most prevalent was bloodstream infection.^(^ ^17^ ^)^ The profile of patients in these settings and the health system organization may partly explain these findings. Intra-abdominal infections often require a surgical team for their treatment, and such expertise is not available in the emergency units that refer such patients to the *Hospital das Clínicas*, contributing to late diagnosis of these infections. Urinary sepsis, probably quite prevalent, is no longer identified upon arrival in the tertiary hospital, since these patients receive antibiotics in the emergency care units, and most use indwelling urinary catheter, factors that hinder the final source diagnosis.

Sepsis with an abdominal source was statistically related to septic shock. Since an abdominal infection source is often subject to surgical drainage, no control of intra-abdominal abscesses can maintain immune stimulation, resulting in septic shock. The high number of positive blood cultures in this site (28%) may indicate the persistence of the origin of undrained infection in the abdominal cavity.

A potential contamination at the time of collection probably resulted in the overestimated of coagulase-negative staphylococci cases in our study. Coagulase-negative staphylococci account for 20 to 30% of infections in critically ill patients, and are usually associated with central catheters and implantable devices.^(^ ^30^ ^)^ When we observe the distribution of pathogens per infectious site, this microorganism predominates in virtually all sites. Since patient data and blood culture results were independent, it is possible that these patients have experienced multiple sepsis episodes, and consequently have several blood culture samples, and this pathogen may, in some situations, be responsible for the infection. However, due to the retrospective nature of this study, it was not possible to assess whether coagulase-negative staphylococci detection was associated with infection or contamination of samples during collection, and these findings need to be analyzed further in prospective studies. Overall, the pathogens isolated from each site were as previously described, predominantly enteric GNB and non-fermenting in respiratory infections, and enteric GNB and enterococci in intra-abdominal infections.^(^ ^27^ ^)^

The retrospective data collection is the main limitation of this study, and some facts are noteworthy:

- An accurate account of the events during hospitalization cannot be obtained from a review of discharge summaries.

- The inclusion of pediatric and adult data may have affected peculiar characteristics of each unit, but the aim of this study was to have an overview of sepsis cases in the organization.

- The updated sepsis definitions reported by Singer et al., were no adopted,^(^ ^1^ ^)^ for the consensus definitions were disclosed after the data collection of the present study. Nevertheless, in order to avoid conflicting findings with the current definitions, we analyzed the results comparing only the groups with presence or absence of sepsis, and for the severity assessment only the cases of septic shock were included.

- Because of the retrospective study design, evaluation of laboratory and clinical data to calculate the score severity as Sequential Organ Failure Assessment (SOFA) were limited to the available data. Even with these biases, the data are consistent with those found in national reports, and highlight the need to improve care for this important and often life-threatening condition.

The impacts of sepsis in an institution are often related to the clinical and epidemiological characteristics of its patients. However, a critical evaluation of the data, and the comparisons among inter-institutional variations are important as they can indicate important differences in (i) the organization and care provisions, (ii) the ability of health care providers, and (iii) resources available. These analyses can be used to formulate local policies for managing this serious public health problem.

## CONCLUSION

The mortality of intensive care unit patients was higher in older patients with sepsis, and with longer hospitalization time. The delay in hospitalization and difficulty in the diagnosis of sepsis can be factors that justify this high mortality. The main infection sites found were pulmonary and intra-abdominal. The low positivity of blood cultures and high frequency of isolated coagulase-negative staphylococci emphasize the importance of implementing good practices of sample collection.
